# An ongoing process: A qualitative study of how the alcohol-dependent free themselves of addiction through progressive abstinence

**DOI:** 10.1186/1471-244X-9-76

**Published:** 2009-11-24

**Authors:** Mei-Yu Yeh, Hui-Lian Che, Shu-Mei Wu

**Affiliations:** 1 Department of Nursing, Chang Gung Institute of Technology, Taoyuan, Taiwan; 2 Department of Geriatric Care and Management, Chang Gung Institute of Technology, Taoyuan, Taiwan; 3 Doctoral Student, Graduate Institute of Clinical Medical Sciences, Chang Gung University, Taoyuan, Taiwan

## Abstract

**Background:**

Most people being treated for alcoholism are unable to successfully quit drinking within their treatment programs. In few cases do we know the full picture of how abstinence is achieved in Taiwan. We tracked processes of abstinence in alcohol-dependency disorders, based on study evidence and results. This research explores the process of recovery from the viewpoint of the alcohol-dependent.

**Methods:**

Semi-structured interviews were conducted in two different settings, using purpose sampling, during 2003-2004. The data were analyzed using content analysis. Participants were 32 adults, purposefully selected from an Alcoholics Anonymous group and a psychiatric hospital in North Taiwan.

**Results:**

We found that the abstinence process is an ongoing process, in which the alcohol-dependent free themselves of addiction progressively. This process never ends or resolves in complete recovery. We have identified three stages in the struggle against alcoholism: the Indulgence, Ambivalence and Attempt (IAA) cycle, in which the sufferer is trapped in a cycle of attempting to give up and failing; the Turning Point, in which a Personal Nadir is reached, and the Ongoing Process of abstinence, in which a constant effort is made to remain sober through willpower and with the help of support groups. We also discuss Influencing Factors that can derail abstinence attempts, pushing the sufferer back into the IAA cycle.

**Conclusion:**

This study provides important points of reference for alcohol and drug service workers and community healthcare professionals in Taiwan, casting light on the abstinence process and providing a basis for intervention or rehabilitation services.

## Background

Alcohol-dependence causes physical and emotional problems and has far-reaching influence in terms of family life, employment, violence and crime [1-3]. Families and society are disrupted. An epidemiologic study found that the prevalence rate of alcohol dependence and abuse among Taiwanese, according to the DSM-III criteria, was 4.9-11% [4]; lower than has been reported in the United States 13% [5]. However, the results of the study [6] in a 1993-1994 epidemiological study in Taiwan suggested that the prevalence rate according to the DSM-III-R criteria in a primary-care setting was 13.8%. Hwu *et al*. [7] said the Taiwan Psychiatric Epidemiological Project revealed a marked increase in alcoholism in Taiwan during the past six decades. A two-year follow-up study of 163 Taiwanese inpatients initially hospitalized for alcohol-dependence found that only around 15.3% recovered completely after detoxification, and the rehabilitation process continued whilst in hospital; whereas around 22.1% died, and the remainder were unable to overcome problematic drinking [8]. Based on this survey, Taiwanese alcohol-use disorder patients are more likely to die than overcome problematic drinking after they receive treatment. Few problematic drinkers can truly maintain long-term recovery from alcohol dependence, and the great majority are unable to remain totally abstinent after treatment.

Among current methods for treating alcoholism, Alcoholics Anonymous (AA) helps sufferers achieve good levels of abstinence through group support. Galanter *et al*. [9] believe that its 12 steps and spiritual orientation play a significant role in the restoration of health and foster change in the sufferer. Thus, attendance of AA meetings is even more important than outpatient treatment. Galanter [10] proposed that AA is a kind of theistic religion, in that alcoholics need a higher power to help them achieve recovery; it can even help atheists discover spirituality in their lives and emphasise relations of partnership. The Betty Ford Institute Consensus Panel [11] argues that recovery is voluntary maintenance of sobriety, individual health and "citizenship;" sobriety means staying off alcohol and all other kinds of non-prescription medicines, its maintenance being a matter for the individual; individual health means well-being in mental, physical and societal terms; and "citizenship" refers to voluntary activities, lifestyle enhancement and contribution to society.

Galanter [10] proposed that recovery is a state of remission, a response to observable and measurable substance abuse. He believed that it is a subjective experience, originating in individual reflection and self-examination, and has connections with "peak experience" proposed by Maslow, reflecting other people's expectations, and strengthening self-esteem and self-realization. White [12] also sees "remission" as only the elimination of AOD-related problems, while "recovery" has a wider meaning, covering "global health"-- "remission" and health in physical, cognitive, emotional, interpersonal, educational/vocational and ontological terms.

Research in Taiwan has rarely taken the perspective of the alcohol-dependent themselves. We have no complete picture of how abstinence is achieved, or why the effort may fail--or what features it shows, and what processes and changes sufferers experience. Accordingly, this research uses qualitative methods to study the process of achievement of abstinence in Taiwan in its entirety. We hope to define and explain behavioural characteristics, and look at influencing determinants, from the viewpoint of the alcohol-dependent themselves. We hope that our results can assist hospitals and community health counsellors, and provide reference points and guidelines for treatment, based on the needs and condition of addicts.

## Methods

This study's main goal was in-depth understanding of the processes of abstinence in alcohol-dependent people, to gain: 1). A complete picture describing the process of achievement or abandonment of abstinence; 2). Understanding of the factors that dictate whether or not abstinence attempts are successful, and behavioural characteristics in successful instances. A qualitative design was used. Research had two stages. In the first stage, we studied cases where AA subjects have successfully given up drinking, to understand the experiences and processes involved. By studying AA subjects who had repeatedly undergone unsuccessful hospital treatment, we aimed to create a more complete picture of the struggle for abstinence and to better understand the causes of failure--this was the second stage. After discussing and checking case histories to get a clear idea of patient condition, we selected subjects in stable condition. In all cases, we used purposive sampling to select interviewees, and used in-depth interviews to gather data.

### Participants

All 32 interviewees (3 females and 29 males) were accessed through AA and the Institutional Review of Taipei City Psychiatric Centre. Informed consent, for both stages, was obtained in advance from each informant. All personal names were removed to ensure confidentiality as promised in the consent form.

#### Stage 1

We primarily used a community setting, with an AA venue in north Taiwan as research field. This group consisted entirely of people with experience of alcoholism. Initially, researchers came to AA meetings as observers, to establish relations of mutual recognition and trust. Then the aims of the research were explained, to win the agreement of the AA members, and subjects were selected by purposive sampling for in-depth interviewing. Individual interviewing followed after each meeting.

Sampling selection criteria: subjects had been sober for more than a year, with sustained full remission. We recruited nine AA members (eight male) with alcohol-use disorder histories ranging from 21 to 31 years. The average age of the AA members was 44.8 years (ranging from 31 to 59 years inclusively). They were all of Han ethnic background. The largest proportion (*n *= 7, 78%) had a basic education (junior high school or high school), and all were employed. Most importantly, these people had been sober for an average period of 62.4 months, with periods of sobriety ranging from 15 months to 105 months.

#### Stage 2

We used a structured therapeutic environment in a hospital psychiatry centre, and all procedures were reviewed and approved by the Institutional Review Board of Taipei City Psychiatric Centre. Based on research design and in coordination with inpatient treatment schedules, the researchers used purposive sampling to select subjects for in-depth interviewing. After all treatments were administered, the researchers went into the conference room with subjects to conduct in-depth individual interviewing. A total of 23 alcohol-dependent inpatients (21 males), ranging from 17 to 30 years inclusively, were recruited in accordance with the sampling criteria: (a) alcohol addicts; (b) those who had been hospitalized to dry out at least once; and (c) those in hospital in a stable condition and able to verbally express themselves clearly. They were also all of Han ethnic background. The largest proportion (*n *= 15, 65%) had a basic education (junior high school or high school), and most (*n *= 16, 70%) were not employed.

### Data collection

Three methods of data collection were used: semi-structured interviews; field notes and AA interviewees' notes. Researchers first conducted pilot study interviews with participants who had successfully given up alcohol for at least one year, to confirm that issues that could be reflected in interviews. Then an interview outline was completed. For the first stage of the study, with nine AA members as interviewees, we interviewed one member based on the outline. This subject said the guideline questions could indeed cast light on the process of stopping drinking, so we did not modify the interview guidelines (Table 1). Afterwards, all nine interviewees expressed the fear that excessive drinking had impaired their ability to remember and that they would be unable to make retrospective statements on the day of interviewing. So, "AA interviewee notes" were made based on questions previously raised in the interview guidelines, to ensure clearer answers at the time of formal interviewing. Questioning focuses were factors influencing stopping drinking, and for maintenance of sobriety and abstinence, so we could create clearer context. With interviewee agreement, we made audio recordings and field notes in formal face-to-face interviews. When verbatim transcriptions of recordings were made, we were able to enrich the content using AA interviewee notes previously provided.

**Table 1:** The interview guidelines

For alcohol-dependent inpatients	For AA members
Would you please talk about your first drinking experience?	How long have you been sober?
What happened the first time you got drunk?	Can you describe the feeling when you were experiencing a craving for alcohol, but eventually overcame it?
What situations lead you to drink?	How do you overcome this craving?
How do long periods of drinking impact upon you?	What made you quit drinking?
Have you ever wanted to stop drinking?	When did you quit drinking?
What do you do to try to stop drinking?	How do you stay sober and avoid a relapse?
What is the result?	

In the second, inpatient, stage, we had to adapt the interview outline to the hospital environment. Pilot studies were conducted on three subjects, before drawing up formal interviewing guidelines (Table 1). We conducted face-to-face interviews, in a relaxed and unforced environment, with 23 subjects. With their agreement, we also made recordings and field notes and asked them to make retrospective statements or explain their motives for hospitalization, in line with interview guidelines. When it was unclear why previous attempts to give up drinking had failed or when answers were vague, we sought clarification of processes, influencing determinants and contexts. Using the information from the two stages, we were better able to construct a clear complete picture of the recovery process.

Each interviewee was interviewed for between 1.5 and 2 hours. All interviews were transcribed verbatim. All procedures were approved by the Institutional Review Board of Taiwan's Taipei City Psychiatric Centre.

### Data analysis

Data were analyzed using content analysis. Interviewee statements about abstinence and addiction behaviour were then subjected to analysis, comparison and categorization. We assigned notes and codes to interviewees' ideas and patterns of thought or behaviour that were particularly important or repeatedly cited. Our study of drinking, alcoholism, withdrawal symptoms, the repeated struggle to stop drinking, and success/failure in abstinence, based on interviewees' viewpoints, yielded a process for abstinence. We found that the indulgence-ambivalence-attempt (IAA) cycle is a process of wavering between addiction and abstinence. Moreover, the data show that sufferers in the Attempt stage have not yet experienced the great Turning Point of self-awareness (Personal Nadir), but can still show abstinence motivation and behaviour. If, in the abstinence process, it is not possible to conquer Influencing Factors, they will lapse back into Indulgence. Therefore, indulgence-ambivalence-attempt stages can be induced in the IAA cycle, revealing a process of repetition.

**Figure 1:**
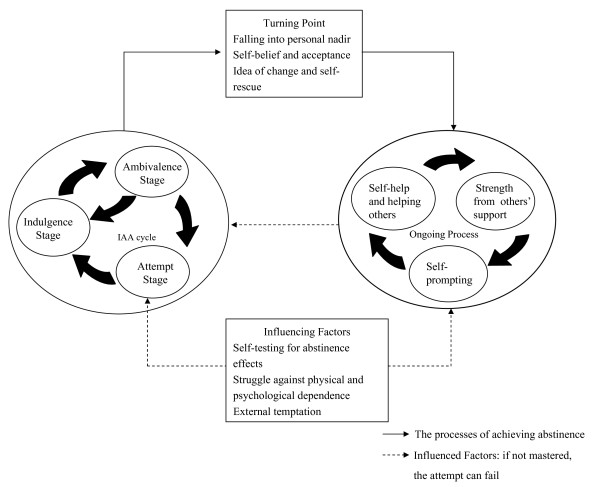
**Progression and no end of abstinence process**: An overview of the processes of recovery of the alcohol-dependent in Taiwan.

Only by going through the nadir experience is motivation for change possible, which is why we refer to it as the Turning Point. It creates opportunities to enter the Ongoing Process stage; those interviewees who had successfully given up alcohol said that those who quit must remain sober indefinitely if they are to return to health, or else failure will result. In addition, in the Ongoing Process, the Influencing Factors (Table 2) can still cause failure and relapse into the IAA cycle. In creating a process for changing behaviour, the researchers, using interview materials and context of the abstinence process, inserted at suitable places the Turning Point, Ongoing Process (maintenance) and Influencing Factors to complete an overview of the processes of recovery of the alcohol-dependent in Taiwan (Figure 1).

**Table 2:** The definitions and quotations of the factors influencing personal decisions to stop drinking

*Influencing Factors*	*Definitions*	*Quotation*
Self-testing for abstinence effects	When the alcohol-dependent persons believe that they no longer need a drink, and when they can completely abstain from alcohol. They try to test themselves, for example, to pick up a glass of alcohol so as to prove that they were successful as regards abstinence. Unfortunately, they fail again.	*"I have not had a drink for a period of four years, and I thought that I no longer had my previous excessive drinking problems, so that I eventually tried to drink to demonstrate the fact that I had quit the use of alcohol completely, but then in the end I was admitted to the hospital after one month."*
External temptation	Alcohol exists everywhere. It is easy to buy it in the convenience store. Once alcohol-dependence individuals' willpower is too weak to resist, or while alcohol is likely to increase interpersonal relationships, they were apt to drink once again, and then relapse into the same bout of excessive drinking.	*"I like to go for a chat with some friends, and, sometimes, I cannot reject the likelihood of an alcoholic drink. Finally, I relapse into alcoholism again; I am unable to stop drinking."*
Struggle against physical and psychological dependence	During the period of stopping drinking, the period of time that one withdraws from the use of alcohol, it causes suffering, and often leads to great physical discomfort, so as to get rid of the uncomfortable impulse that causes us to drink. As regards psychological factors, caring, having emotive disturbances, or interpersonal relationships problems or anything involving a level of stress, alcohol-dependent individuals were simply given to drinking to escape, rather than a need to deal with them.	*"When you are able to stop drinking for a long time, but then, you feel physical discomfort as a result of not drinking, it is really very comfortable that the first cup can be drunk, and it all becomes a very relaxed feeling all over."* *"I should be able to say that I see alcohol but that I don't necessarily drink it, but when I am facing the stress of living, or perhaps in a bad mood, I may start to drinking again, and in that way, it would let me escape such states and trouble."*

### Rigour

Research rigour criteria, such as those proposed by [13,14], were met through several strategies. With regard to credibility, the first author, a psychiatry expert, had prolonged engagement with participants, and built up relations of trust, so that during collection of data, it was possible to get closer to the true feelings and thoughts of participants. For transferability, in-depth interviewing in natural conditions enabled a wealth of information to be obtained, leading to rich descriptions. Confirmability and dependability were assured through checking and coordination with the literature, study groups, data analysis memos, and regular research group meetings, assuring objectivity and neutrality. Member checks were done with interviewees (for example, two AA subjects); we showed them our findings and complete picture to get their feedback.

## Results

Figure 1 shows the abstinence process, including the IAA cycle, Turning Point and Ongoing Processes. Influencing Factors are included to give a complete picture.

### The IAA cycle

IAA is short for the Indulgence, Ambivalence and Attempt stages. In Indulgence Stage, alcohol-dependent individuals and their families recognised that the sufferers had virtually no control over their consumption at all. Physical and mental impairment was fundamental. As they attempted to overcome withdrawal discomforts, they became, paradoxically, more alcohol-dependent. Interviewee 26 said,

"When I had physical problems and saw the doctor, they never got better. But I felt good when I had a drink. I started relying on alcohol and started wanting to drink all the time. Drinking would help me feel better."

When there is no control over alcohol, and physical condition is deteriorating, alcoholics and/or their families perceive the issues underpinning their motives for quitting. They may have joined AA or simply stopped drinking by then and/or accepted treatment in hospital. At this stage, the 32 interviewees moved, at their own pace, into the Ambivalence stage.

In Ambivalence Stage, they want to quit, but, still more, they want to drink. Alcohol-dependent persons struggle to make up their minds to give up drinking. They are afraid. It is usually difficult to resist the craving. Interviewee 25 said,

"I'm afraid of life without alcohol. I've been around alcohol for so long it's become a part of my life. Oh! If I had to give it up all at once, I'm really afraid of that kind of bleak existence."

They feel that they still have some control, using their will, and believe that drinking isn't a problem for them, yet. Interviewee 24 said,

"Other people keep pointing out my problem and I can't accept that. I'll admit I have a drinking problem, but I think I can control it."

When first joining AA, some sufferers reject the "Western religious ritual" of admitting that they are alcoholics, and often cannot publicly admit it either. Only two interviewees lingered between the Indulgence and Ambivalence stages (IA cycle, Figure 1) and could not move into the next stage. They would fall back to the Indulgence stage, especially when faced with physical or psychological stress. The other 30 interviewees so feared and felt physical and psychological pain that they eventually determined to overcome their drinking. Once they eventually were able to abstain, they had moved into the Attempt stage.

In the Attempt stage come recognition of deteriorating physical condition and family relationships, and resolve to change behaviour.

In effect, sufferers chose some way(s) to quit, by themselves, and/or seeking treatment in hospital and/or joining AA again. Interviewee 12 said,

"If somebody were drinking over here, I'd just take a different route. I wouldn't pass by anywhere where everyone knows me. We drank together. We know each other. If there's alcohol on this street, this is a street I won't walk down."

However, they often struggle with the craving. They have to go through the frustrating experience of alcohol dependence, over and again, to keep motivated. Escape is possible if they have support from their families and the AA, and, in many cases, a steady job and normal lifestyle. Twenty-one interviewees had successfully given up drinking for a period, but they were unable to overcome the three Influencing Factors and eventually relapsed (IAA cycle, Figure 1). Interviewee 9 said,

"After I sobered up, it was all the same. I still didn't feel good. I still wanted to hurry to get back to drinking again. It was a cycle; recurring over and over again."

Nine interviewees (AA subjects) who clearly realised the chaos and desperation of their situation fell back into the Indulgence stage. They said, "my life is at its lowest ebb", and the feeling was of "having fallen to a personal nadir" (hopelessness, feeling of uselessness or impotence). They then moved from the IAA cycle to the Turning Point. Twenty-one of the 32 interviewees remained in the IAA cycle, and the two of the twenty-one interviewees are still in the Indulgence and Ambivalence stages (IA cycle).

### Turning Point

The Turning Point is crucial for alcohol-dependent persons, who now show both destructive and reconstructive tendencies. It has three characteristics: the Personal Nadir, self-belief and acceptance, and embracing the idea of change and self-rescue.

All of the nine AA interviewees suffered extreme physical and psychological pain, and worse than before in the Indulgence stage. These people were again totally controlled by alcohol. Their family situations were extremely chaotic, interviewee 26 said everybody sinks to a Personal Nadir, but this experience is not the same for everybody. If they have not reached that lifetime's Personal Nadir, no drying-out treatment can succeed. She recalled her own Personal Nadir:

"When drinking, I suffered physically and everything was controlled by alcohol. My personal relationships were destroyed, and I could not see where I had gone wrong, so I felt that it was the other person's fault, or my environment, I felt God was not being fair to me, and so I drank. In the end, I was in a state of mental collapse, and I didn't know what to do. I was floating on the verge of death."

In their despair, alcohol-dependent persons may intend suicide through drinking, but mostly, they are also afraid of dying. They "try to commit suicide by alcohol, but just cannot make it." Interviewee 24 recalled,

"I drank until it was really agonising, even when I wanted to give up drinking. My wife said, 'It's up to you if you want to drink yourself to death.' In my drinking days, I really did want to drink to finish my life."

With regard to self-belief and acceptance, alcoholics must admit to themselves that they are drunkards--desperate, often hopeless--and have chaotic lives. They have to realize their condition and search for help, continually, through support groups (for example AA). Interviewee 26 said, "Alcohol is crafty; it's stubborn. It won't quit just because you quit. It sneaks up and catches you."

At this point, they have ideas of change and self-rescue. But the nine AA interviewees realised that they were not able to complete or endure the abstinence process alone; they were still vulnerable in their state of mind, and needed help from support groups. Alcoholics are often motivated; they "want to live" and "want to win," and abstinence is the only hope. Interviewee 26 emphasised that, "I didn't drink myself to death in the end, so I've got to keep on living as best I can."

If alcohol-dependent persons can exhibit resolve, their lives will be turned around. They can take the key step into the Ongoing Process, finding ways to continue abstinence and get support.

### Ongoing Process

There are three aspects to this: strength from others' support, self-prompting, and self-help and helping others and all are essential for the success of any attempt to remain abstinent. It is an indefinite process. Support comes from self-help groups and families. In the self-help groups, sufferers exchange experiences, communication and hopes. Recovering alcoholics pool resources. They go to AA meetings every day, and offer and receive respect and concern. When they have emotional problems, they turn to counsellors, pray, or read the Anningjing (Serenity Prayer).

In addition, family members who stand by in the background, and who give encouragement or accompany sufferers to self-help group meetings, are also a motivating force. Interviewee 23 said,

"During that time when I just beginning to go to AA, my sisters took turns to take me there...... one came on Monday, Wednesday, and Friday, the other on Tuesday, Thursday, and Saturday. After work, they came to my company and took me to the meetings...... At that time I had to also muster a great deal of courage, I wanted my family members to see me dry out."

Interviewees were asked to carry out a "recovery plan" to win praise and develop more positive attitudes. They had to constantly be on their guard against the Influencing Factors, and avoid "taking a glass." Interviewee 24 said that alcohol is like a monster that always waits for a chance to attack you.

"This group has strategies like, 'No matter what, don't take a glass of alcohol,' because if I take it, I'll just completely lose control."

Self-help and helping others not only mean the alcohol-dependent can get support, but also, through sharing of individual experience with new members, can help other people. At the same time, they can learn from the experiences of new members, maintain their own sobriety and ensure that they do not fall back into the IAA cycle. Interviewee 24 said, "Helping people is the best way to stay vigilant myself." Alcoholics can get their lives on track and, eventually, regain their freedom if they consistently resist drinking. Interviewee 22 said,

When I was in abstinence periods, the craving would always be there. I don't know when, but, eventually, I became a completely free man. Now, I don't avoid convenience stores or street vendors that sell alcohol.

In this process, alcohol-dependent persons have stayed dry, and changed their mindset. They can handle their emotional problems and interpersonal conflicts, and show gratitude. They have found balance in life, without alcohol, and stay sober.

## Discussion

### Repeat cycle processes

This research shows that the process of giving up alcohol is one of repeated cycles. By compiling a complete picture of this process, based on the shared experience of participants, we can identify the IAA, Turning Point and Ongoing Process. This complete picture is characterised by "direction" and "relapse." "Direction" refers to the process of stages that those who wish to successfully give up alcohol must go through, and "relapse" refers to the tendency, when unable to overcome the Influencing Factors, to relapse after successfully quitting or lapse in the attempt; those attempting abstinence at the Ongoing Process or Attempt stages may revert to the IAA cycle, and repeat the process over and over again. Our complete picture of the process of achieving abstinence is very similar to Prochaska and DiClemente's trans-theoretical model (TTM).

In 1982, Prochaska and DiClemente used the trans-theoretical model (TTM) to attempt to integrate 15 different theoretical constructs, into a single comprehensive framework, hence the name trans-theoretical. In addition to the central construct of stage of change, the model comprises the 10 processes of change. The version of the model used most widely in recent years specifies five sequential stages: pre-contemplation, contemplation, preparation, action, and maintenance [15]. Our findings regarding the IAA cycle are similar to the stages of pre-contemplation, contemplation, preparation and action etc; For example, pre-contemplation and contemplation are similar to the Indulgence and Ambivalence stages, and action is similar to the Attempt stage. Our Ongoing Process is similar to maintenance, when sufferers cannot resist the temptation of alcohol and relapse. But we found the Turning Point is the critical point for the sufferers, deciding whether or not they will succeed in abstinence. Influencing Factors are the determinants for relapse; and our findings reveal there is no time limitation in any stage. However, there are recovering alcoholics with at least 1 year of abstinence in the Ongoing Process, and they are still vulnerable. It is necessary to remind them to overcome the Influencing Factors. Here, our study differs from TTM.

Prochaska and DiClemente [16] have indicated that health-behavioural changes are a dynamic process, and that the behavioural changes appear to occur in a cyclical model, and that the changes often occur repeatedly throughout the processes. Movement throughout these stages is not necessarily a straight forward pathway from the initial pre-contemplation to maintenance stage [17]. The pattern of successful change is typically conceptualised to be spiral, with relapses to earlier stages and re-cycling through the stages, typically occurring prior to final remission (if successful), or the long-term maintenance stage, is attained [15,17-19]. The trans-theoretical model (TTM) implies that changes in healthy behaviours move in a spiral fashion. This study finds that an addiction cannot be changed within just one cycle. The behaviours move partway through the cycle and then revert to a lower position. Alcohol-dependent persons repeatedly move between drinking and abstinence. The changing process is a dynamic process that repeats over and over again. Looking at the processes of behavioural change from a western cultural perspective, such processes among Taiwanese take different approaches but have similar goals, showing that the process of reforming addictive behaviour is basically similar for all people around the world.

### Lapse management strategies

Influencing Factors can decide whether abstinence is successful or not. If alcoholics overcome them they stay dry in the Ongoing Process; if they cannot overcome them, they fall back into the IAA cycle. Alcohol-dependent individuals in the Indulgence or Ambivalence stages are not very aware of Influencing Factors. They are torn between abstinence and craving. In the IAA cycle, frustrated sufferers may try to overcome alcohol, but their resolve may be vulnerable [20]. In the process of maintaining abstinence from alcohol, strong willpower in the face of the temptation, and the mere idea, of drinking, is essential. However, the craving can come at any time and challenge resolve at the Attempt and Ongoing Process stages. Therefore, to conquer the Influencing Factors, vigilance and self-prompting are constantly necessary. A relapse into the IAA cycle results in an even stronger alcohol dependence and deeper crisis.

This study shows that in the process of relapse, the Influencing Factors are the key determinants. Influencing Factors include situations where recovering alcoholics are placed in high-risk situations; if they can adopt effective self-efficacy strategies, then they can avoid lapse or relapse. For example, the subjects of the study in AA used a tactic of constantly prompting themselves, or they talked to counsellors about their craving and struggle against the urge to drink. Based on counsellors' accounts of their own past mistakes, they constantly warned themselves and maintained a personal dialogue, to strengthen their self-efficacy and achieve behavioural change, so that the process of health restoration becomes permanent and does not lapse.

The findings of this study echoed those of [21] in their study of the relapse prevention model (RP model), in that when recovering alcoholics are in high-risk situations, an effective coping response can increase their self-efficacy. Moreover, there is a decreased probability of relapse. If the coping response is ineffective, it can lower self-efficacy and positive outcome expectancies for effects of alcohol, leading to failure in the abstinence attempt. The abstinence violation effect therefore increases the probability of relapse.

The relapse prevention model uses cognitive and behavioral strategies to prevent and limit occurrence of relapse. The authors suggest that individuals' coping behaviour in high-risk situations can be decisive. For example, if the behavioural strategy is removing yourself from the risky situation or avoiding problematic places/events, and cognition strategy is positive self-dialogue, this can reduce the occurrence of relapse. Therefore, skills training, cognitive restructuring and lifestyle balancing are strategies for prevention of relapse [21].

During the skill-teaching process, it is necessary to teach recovering alcoholics how to change their habits, rather than use alcohol to test their individual willpower [21]. This is consistent with the results of our study. To pick up a glass of alcohol is a test of the success of the attempt to give up alcohol, and also of the will. It cannot succeed in promoting sustained abstinence. In terms of cognitive constructs, it is necessary to avoid the idea that a lapse is a personal failure. The recognition should be that it is a mistake, so as to reduce feelings of guilt and failure--a point also rose by AA members taking part in our study. After a lapse, it is still necessary to go to AA meetings and draw on the support of the group, to understand that the erroneous behavioural choice was the result of failure to fully transform drinking behaviour. Stability in lifestyle, relaxation training, stress and time management can all help stabilize lifestyle [21]. AA members in our study emphasized that an orderly life, stable job and emotional management are important factors enabling them to normalize their lives. This shows that behavioural and cognitive strategies were of use to recovering alcoholics in our study.

It is necessary to ensure that recovering alcoholics realize that relapse is an ordeal they cannot avoid. In particular, the role of Influencing Factors is crucial. As [21] argue, this research agrees that the occurrence of urges and craving are brought about by psychological or environmental stimuli. To achieve sustained and successful abstinence, it is necessary to have a coping strategy for these Influencing Factors and enhance self-efficacy and empowerment.

### There is no end to the abstinence process

In the Ongoing Process, alcohol-dependent people succeed in quitting, and remain abstinent. This endless process, our research found, requires constant vigilance. Moreover, achievement is only possible by drawing on group and family strength. This study found that sufferers at the Ongoing Process stage have a more positive attitude to life; once free from the alcohol trap, they are free again and can get work and family life back on track. Some scholars suggest recovery is a process. Addicts who have beaten their addiction can regain a positive attitude to life, including the feelings of their family, and have healthier work patterns and make more contributions to society than before they became addicts [10,12].

The three aspects in the Ongoing Process are cardinal. Giving up drink is not something one can do alone; support is needed. However, Murray *et al*. [22] argue that members attending AA meetings found no influence of the "God belief" and "higher power" as motivators to stop drinking and stay sober. There is a need for an operational definition to be applied to the spiritual aspect of recovery. Our study is distinct in that it is based on the perspective of recovering alcoholics themselves. They believe that conquering alcoholism is not simple, and when craving reappears, they must contact counsellors and use the Serenity Prayer, or turn to the higher power to get through the torment. Our study findings are similar to those of Yeh *et al*. [23], family and uninterrupted participation in an abstinence group are the support.

This would appear to be identical to the findings of [24,25] regarding the role of self-help groups in maintaining abstinence, and is probably relevant to findings regarding self-help groups in which members share experiences, tell their own stories, learn how to refuse alcohol at social gatherings and describe their personal freedom from alcohol [25], and to the sense of belonging and affirmation provided by self-help groups [25,26]. McCrady *et al*. [27] suggested that clinical professionals should be familiar with and introduce self-help groups to alcohol-dependent persons when they are first hospitalized, to encourage inpatients to join them and gain a better chance of recovery [28]. Furthermore, our findings agree with [29] in that helping other substance abusers is also a good way of maintaining one's own sobriety. Cohorts' supportive attitudes can bring greater meaning to individual lives. However, Yeh *et al*. [23] found that successful recovery from alcohol dependence was an empowerment process, and, through self-awareness, sufferers must create motivation and constantly prompt themselves to achieve enduring abstinence.

The literature [30,31] shows that the AA 12-step facilitation program is a suitable strategy for most individuals with regard to aftercare programs. However, our study found that AA, originating in the west, has both positive and negative effects from the Taiwanese participants' perspective. Those participants who could accept AA in this study were usually persons with severe addictive problems. In the Personal Nadir and Turning Point, the alcohol-dependent find motivation to resist and seek a chance "at a new life." They are more likely to spontaneously reach the conclusion that giving up alcohol is beyond the powers of the individual and has to be achieved through group help and strong spiritual faith. They accept that they are alcoholics and achieve complete humility, and develop a willingness to join AA, in the end creating opportunities for success in conquering alcohol problem.

By contrast, the other participants who cannot reach the Ongoing Process are reluctant to admit that they are drunkards, and cannot accept the religious rituals arranged by self-help groups due to Eastern and Western cultural differences. Based on interview data, a few participants believe that they have not yet reached the point of inability to control alcohol abuse, and find it hard to recognise themselves as drunkards. To be called a drunkard means to be despised, and to be thought to have no sense of dignity. In traditional Chinese culture men are treated with more respect than women, men have relatively higher status in family. When they join AA, alcohol-dependent individuals must to admit they are drunkards through an AA ritual. This creates a sense of being belittled and despised. Furthermore, the predominant religions in Taiwan are Buddhism, Taoism and folk beliefs. To join AA, it is necessary to accept western religious beliefs. Some participants who said it is relatively difficult to accept faiths you are unused to or which go against individual or the household religious tradition. They cannot accept AA values and withdraw from the program after initial participation. Our study revealed the experience of participants' abstinence, and focused on the third of AA's 12 steps--make a decision to turn our will and our lives over to the care of God as we understood Him, an important spiritual element for Western Christianity or other religions. However, self-help groups may need to adapt Western AA to the context of Chinese culture and individuals, using the term higher power- "Buddha's blessing" instead of "God," which is more acceptable to Chinese individuals.

This is critical in the Ongoing Process. It would appear to echo exactly the findings of [32], who postulated that alcohol-dependent persons are often aware that their problematic drinking has caused social problems and exhausted their and their families' resources, in addition to causing desperation and stress. Usually the alcohol-dependence resort to therapy when they reach their Personal Nadir. In the abstinence experience of the nine AA interviewees, this study finds that the alcohol-dependent decide to give up drinking when they lose control over their lives and feel desperation at their deteriorating physical condition and family relationships.

Finally, some of those who lapse also lack confidence and cannot commit themselves to abstinence. They promise only to reduce consumption, but cannot always control themselves once they start drinking again. Many are destined to struggle, repeatedly, within the IAA cycle, and many are hospitalized for detoxification many times.

In general, alcohol consumption or alcohol drinking behaviour is more prevalent in Western countries than in Taiwan. Tea is a main part of daily life rather than alcohol. Furthermore, Yang [33] study revealed that in Chinese society, drinking is used as a means to promote social relations and communication, and alcoholic beverages are consumed only at parties or during mealtimes. Many Chinese drink alcohol mainly at special occasions such as weddings, celebrations, festivals, or business negotiations. Drinking alcohol is perhaps part of social behaviour, and refusing alcohol is seen as a disrespectful or giving no face to others. Based on participants' recollections, this was why they had relapsed into alcohol abuse again. Some demographic data of the participants such as occupation or social-economic status revealed that AA participants in employment have stable work and are maybe more likely to refuse alcohol than alcohol-dependent inpatients. Therefore, alcoholics and their family members believe that full prevention of drinking is hard to achieve, but if the goal is reducing volumes of alcohol consumed, this should be relatively easy to accept and achieve. This is also a reason underlying self-rationalization of hospital inpatients.

However, the results of our studies show that those that wish to give up alcohol must abstain completely. They cannot take a single glass. Harm reduction is not easily effective in achieving successful abstinence for AA individuals. This does not agree with the arguments of [34], who uphold the harm reduction method combined with traditional substance abuse services. They believe that people who have not been helped by traditional substance abuse services can use the harm reduction treatment services to achieve effective treatment. Harm reduction can also be considered as a process aiming for the goal of total abstinence. It might be more effective to engage some individuals for treatment initially by using harm reduction strategies.

Based upon these case studies, we hope psychiatric professionals can understand alcohol-dependent individuals, and assess how their problems are related to their addiction. We hope that the origins of their problems can be identified. An inpatient (Interviewee 12) said, "Every day the nurse takes some time out to chat with the patients, about what they're thinking, what suggestions they have; it would appear that she is collecting information. It's good [that they take time to understand us]."

### Limitations of the study

The generalisability of the study findings is limited by using only 32 alcohol-dependent persons, and only three women. In clarifying how alcohol individuals reach the Ongoing Process, it is based on only 9 AA interviewees. Our future research could study individuals in the community (non-AA), casting light on their pathway to successful abstinence, and whether successful individuals have different abstinence characteristics. The three Influencing Factors play the key roles in the alcohol-dependent persons' progress in processes of change; as mentioned above, they facilitate or obstruct. We believe further research is needed into the questions of what strategies to overcome the Influencing Factors in cases of lastingly successful quitting, and how these Influencing Factors result in reliance on alcohol again in the case of persons who fail. We hope coping methods for designing lapse management strategies at the local level, or harm reduction perspectives, can be included in research into inpatients undergoing treatment. Finally, as individual subjects stated, the Personal Nadir is not the same for everybody. Future research could focus on predicting the timing of the Personal Nadir, and help addicts find ways out of it, since this is the ideal opportunity to intervene with abstinence measures.

## Conclusion

In summary, this research into the complete picture of the process of abstinence from alcoholism finds that changes in addictive behaviours do not necessarily follow a precise timetable. Nevertheless, these changes are apparent with their respective characteristics. Alcohol-dependent individuals are often torn between relapse and sobriety. They have to go through a strenuous Turning Point experience to dry out permanently. It is necessary to be aware that once drinking becomes a habit, it is extremely difficult to overcome. Three Influencing Factors are high-risk situations--self-testing for abstinence effects, the struggle against physical and psychological dependence and external temptation--have to be stressed. The three Influencing Factors play a critical role as to whether sufferers can find balance in their lives without the use of alcohol and uphold the Ongoing Process in its three aspects (self-help, group support and self-prompting). Support is crucial. This study is, therefore, an important reference source for psychiatric professionals trying to understand alcohol-dependent individuals' process of abstinence and providing rehabilitation services.

## Competing interests

The authors declare that they have no competing interests.

## Authors' contributions

MYY was responsible for the study design, obtained funding, supervised the study, and data collection. SMW performed the data analysis and was responsible for writing the drafts of this paper. MYY, HLC, and SMW were responsible for the revising it critically for important intellectual content. All authors read and approved the final manuscript.

## Pre-publication history

The pre-publication history for this paper can be accessed here:

http://www.biomedcentral.com/1471-244X/9/76/prepub
